# Evaluation of a Novel Quantitative Test for Glucose-6-Phosphate Dehydrogenase Deficiency: Bringing Quantitative Testing for Glucose-6-Phosphate Dehydrogenase Deficiency Closer to the Patient

**DOI:** 10.4269/ajtmh.18-0612

**Published:** 2018-10-22

**Authors:** Sampa Pal, Pooja Bansil, Germana Bancone, Sevan Hrutkay, Maria Kahn, Gornpan Gornsawun, Pimsupah Penpitchaporn, Cindy S. Chu, François Nosten, Gonzalo J. Domingo

**Affiliations:** 1 Diagnostics Program, PATH, Seattle, Washington;; 2 Shoklo Malaria Research Unit, Mahidol–Oxford Tropical Medicine Research Unit, Faculty of Tropical Medicine, Mahidol University, Mae Sot, Thailand;; 3 Centre for Tropical Medicine and Global Health, Nuffield Department of Medicine, University of Oxford, Oxford, United Kingdom

## Abstract

Glucose-6-phosphate dehydrogenase (G6PD) deficiency, a common genetic blood condition, can result in kernicterus at birth, and later in life as severe hemolysis on exposure to certain infections, foods, and drugs. The unavailability of point-of-care tests for G6PD deficiency is a barrier to routine curative treatment of *Plasmodium vivax* malaria with 8-aminoquinolines, such as primaquine. Two quantitative reference tests (Trinity Biotech, Bray, Ireland and Pointe Scientific, Canton, MI; Cat No. G7583) and the point-of-care STANDARD^™^ G6PD test (SD Biosensor, Suwon, South Korea) were evaluated. The STANDARD G6PD test was evaluated at multiple temperatures, in anticoagulated venous and capillary samples, including 79 G6PD-deficient and 66 intermediate samples and across two laboratories, one in the United States and one in Thailand. The STANDARD test performed equivalently to a reference assay for its ability to diagnose G6PD deficiency (< 30% normal) with a sensitivity of 100% (0.95 confidence interval [CI]: 95.7–100) and specificity of 97% (0.95 CI: 94.5–98.5), and could reliably identify females with less than 70% normal G6PD activity with a sensitivity of 95.5% (0.95 CI: 89.7–98.5) and specificity of 97% (0.95 CI: 94.5–98.6). The STANDARD G6PD product represents an opportunity to diagnose G6PD deficiency equally for males and females in basic clinical laboratories in high- and low-resource settings. This quantitative point-of-care diagnostic test for G6PD deficiency can provide equal access to safe radical cure of *P. vivax* cases in high- and low-resource settings, for males and females and may support malaria elimination, in countries where *P. vivax* is endemic.

## Introduction

Glucose-6-phosphate dehydrogenase (G6PD) is an essential enzyme that protects red blood cells from oxidative damage caused by certain drugs, diseases, and foods.^1,2^ The X-linked human G6PD gene is highly polymorphic with many mutations resulting in reduced enzyme activity in red blood cells or G6PD deficiency. Exposure to oxidative agents can induce hemolysis in red blood cells with low G6PD activity levels and cause severe anemia, sometimes requiring blood transfusion or causing irreversible renal damage and even mortality, if not managed promptly. Glucose-6-phosphate dehydrogenase deficiency presents clinically in the neonate as jaundice resulting from hyperbilirubinemia; this may lead to kernicterus, a form of brain damage.^3,4^ Several medications including rasburicase- and 8-aminoquinoline–based antimalarial drugs, such as primaquine, are known to cause clinically significant hemolysis in G6PD-deficient individuals. A high of dose primaquine (a 7-or 14-day regimen) is required to cure patients of *Plasmodium vivax* malaria. If a patient is not cured of *P. vivax*, they are at risk of relapse with increasing risk of morbidity and further transmission of the parasite.^5,6^ Relapse contributes to more than 50% of the disease burden in a community.^7,8^ A single dose of tafenoquine, another 8-aminoquinoline, when given with chloroquine is capable of curing a patient of *P. vivax*.^9^ Tafenoquine, recently approved by the United States Food and Drug Administration (FDA) for radical cure of *P. vivax*, under the Krintafel label, is indicated for individuals with greater than 70% G6PD activity. Tafenoquine has also been approved by the FDA with a different dosage for prophylaxis, under the Arakoda label, with similar indications for G6PD deficiency.

Many countries will struggle to meet their target malaria elimination goals without broader safe access to radical cure of *P. vivax*. Diagnostic tests that determine a patient’s G6PD status are needed at or near where they seek treatment.^10^ Glucose-6-phosphate dehydrogenase deficiency is determined by measuring G6PD activity, adjusted for temperature, in blood normalized for either red blood cell count or hemoglobin. Quantitative testing for G6PD is performed in reference or specialized laboratories using a complex assay on a temperature-regulated instrument because of the large temperature impact on enzyme activity. The most commonly used test for clinical screening is the qualitative fluorescent spot test, which accurately discriminates hemizygous-deficient males and homozygous- or heterozygous-deficient females, who typically have G6PD activity less than 30% of normal. Although this is adequate for males who are either deficient (< 30% normal activity) or normal (> 80% activity), it is inadequate to classify females who can be G6PD deficient, intermediate (30–80% activity), or normal.^11–15^

Quantitative point-of-care G6PD tests that can be used in low-resource settings can impact health on many levels, including reductions in neonatal morbidity and mortality. It can also impact health by providing access to safe, radical, curative treatment for *P. vivax* malaria, which can then prevent relapse burden, onward malaria transmission, and accelerate malaria elimination.^7–10,16^ Both qualitative and quantitative G6PD deficiency tests that can be performed closer to the patients are beginning to emerge.^17–19^

In 2017, the production of a commonly used reference assay for evaluating new G6PD products, the G6PDH quantitative test by Trinity Biotech, was suspended.^20^ This study presents the results of a bridging evaluation of the Trinity Biotech kit against a commercially available U.S. Food and Drug Administration–cleared reference assay by Pointe Scientific. The innovative, point-of-care STANDARD G6PD test by SD Biosensor was also evaluated against the same reference assay.

## Materials and methods

### U.S.-based study.

U.S. donor blood specimens were obtained from Bioreclamation, Inc. (New York, NY), which has donor centers in Miami and New York. Specimens, both venous and capillary, were collected between July 2017 and January 2018 from volunteers of African American origin, at least aged 18 years, and who had signed consent under protocol 2010-017 (Western Institutional Review Board, Washington). Study participants were recruited consecutively and with no knowledge of G6PD status. Participants of African American origin were recruited because of the high prevalence of G6PD deficiency in this population. The sample size was calculated to capture a minimum of 20 G6PD-deficient individuals based on previous studies in a similar population.^13^ Specimens of 5 mL volume were collected in dipotassium ethylenediaminetetraacetic acid (K_2_EDTA)−coated anticoagulant venipuncture vacuum tubes (BD Vacutainer^®^; Becton, Dickinson, Franklin Lakes, NJ). Capillary specimens, 0.2 mL were collected in K_2_EDTA vacutainers (RAM Scientific, Nashville, TN). All specimens were transported on cold packs and stored at 4°C until tested. Specimen processing occurred within 2 and 4 days of blood collection. Tests for a given comparison were conducted on the same day. All G6PD assays were performed independently and blinded to the G6PD status. A flow chart describing the sampling and testing performed is provided as Supplemental File 1.

### Thailand study.

An additional study was conducted by Shoklo Malaria Research Unit (SMRU) in Mae Sot, Thailand, involving samples obtained from two clinical sites located north and south to Mae Sot that serve a migrant population composed of Burman and Karen ethnic groups living along the Myanmar border (Tak Province, northwestern Thailand). Adults with known G6PD status were invited to join the study to achieve a convenience sample of approximately 50 G6PD-deficient volunteers (male and female), 50 G6PD-heterozygous female volunteers, and 50 G6PD-normal volunteers.^21^ A total of 0.5 mL of venous blood was drawn from the arm, transferred to K_2_EDTA tubes, and inverted 10 times; samples were kept at 4°C until analysis. Ethical approval was obtained from the ethics committee of the Mahidol University Faculty of Tropical Medicine (FTMEC MO/15/259) and the University of Oxford Tropical Research Ethics Committee (OXTREC 563-15). Written informed consent was obtained from all participants. The blood collection was performed at the clinics.

### G6PD activity by reference assays.

#### Trinity Biotech quantitative G6PD assay.

Glucose-6-phosphate dehydrogenase activity assays were run in duplicate using the quantitative G6PD kit from Trinity Biotech (Cat. No. 345-B) according to the manufacturer’s instructions.

#### Pointe Scientific quantitative G6PD assay.

Glucose-6-phosphate dehydrogenase activity assays were run in duplicate using the quantitative G6PD kit from Pointe Scientific (Cat. No. G7583) according to the manufacturer’s instructions. Normal, intermediate, and deficient controls from Analytical Control Systems, Inc. (Fishers, IN; Cat. Nos. HC-108, HC-108IN, and HC-108DE, respectively) were run using the same method on each day of testing. The G6PD values were corrected using the temperature correction factor (1.37) at 30°C provided by Pointe Scientific.

For both reference assays, enzyme activity was determined using the UV-1800 temperature-regulated spectrophotometer, set at 30°C, by measuring the change in absorbance rate at 340 nm over 5 minutes. Glucose-6-phosphate dehydrogenase activity values were calculated in U/g hemoglobin (Hb).

#### Hemoglobin measurement.

Hemoglobin concentration was determined using the HemoCue^®^ Hb 201+ System (No. 121721, Cat. No. 22-601-007; Fisher Scientific, Waltham, MA) for the U.S. specimens or by automated hematology analyzer (CelltacF MEK-8222J/K; Nihon Kohden, Irvine, CA) for the Thailand specimens.

### Glucose-6-phosphate dehydrogenase activity by the SD Biosensor STANDARD G6PD test.

The SD Biosensor STANDARD G6PD test is designed to measure the quantitative concentration of total hemoglobin (g/dL) and G6PD enzymatic activity (U/g Hb) in fresh human whole blood based on reflectometry assays. The STANDARD G6PD test consists of two components: a G6PD analyzer instrument and a G6PD test kit (Supplemental File 2). The test kit contains the G6PD test devices (strips), individual extraction buffer tubes, SD Ezi tube+ sample collectors, a single lot-specific code chip, and an instruction manual. Glucose-6-phosphate dehydrogenase activity normalized by hemoglobin was measured with the SD Biosensor STANDARD G6PD test, as per the kit instructions.

Briefly, a device/strip is inserted into the analyzer. Next, using the SD Ezi tube+ sample collector, 10 µL of capillary or venous K_2_EDTA specimen is transferred to the extraction buffer tube, which is followed by thorough mixing with the SD Ezi tube+ sample collector. A new SD Ezi tube+ sample collector is used to draw a 10 µL mixed specimen and extraction buffer to apply to the test device. After 2 minutes, the analyzer displays the hemoglobin measurement in g/dL and the quantitative ratio of G6PD to hemoglobin (U/g Hb) simultaneously. The STANDARD G6PD test is designed to require only 10 μL of blood, function over the operating temperature range of 15–40°C, and has a dynamic range for specific G6PD activity of 0–20 U/g Hb. The device used for this study has a total hemoglobin range of 7–25 g/dL, more recent versions have a 4–25 g/dL dynamic range claim.

SD Biosensor also provides unitized high and low G6PD controls to support quality assurance of the STANDARD G6PD test. The control reagents are formulated as tablets, which can be applied to the lysis buffer and then run as a normal blood sample. Both levels of G6PD quality control material specific to the STANDARD G6PD test were evaluated. Level 1 control ranges 0–3 U/g Hb G6PD and 4.0–12.0 g/dL of Hb. Level 2 control ranges 6–10 U/g Hb G6PD and 13.0–17.0 g/dL of Hb.

For the data presented here, all blood transfers and dilutions were performed exclusively with disposable SD Ezi tube+ sample collectors as per the instructions and would be used in typical clinical settings. All STANDARD G6PD tests were performed blinded to the reference assays results.

### Statistical methods.

Correlation between the reference assay and the investigational device assay was measured by linear regression and the bias was analyzed using Bland–Altman plotting. The clinical performance of the SD Biosensor STANDARD G6PD assay against the Pointe Scientific G6PD quantitative spectrophotometric assay was determined by calculating sensitivity and specificity. Sensitivity and specificity of the STANDARD G6PD assay were calculated as described previously.^20^ In summary, an adjusted male median was calculated for the Pointe Scientific spectrophotometric gold standard test and defined as 100% activity from which the threshold activities were determined. Area under the curve (AUC) of the receiving operating characteristic (ROC) curve was calculated at different activity thresholds to analyze clinical performance of the STANDARD G6PD test.^22^ Youden’s index was calculated to determine the optimal cutoff point at various thresholds.^22^ All statistical analyses were conducted in Stata 13.0 (Statacorp, College Station, TX).

## Results

Performance data are presented for three G6PD activity thresholds: 1) < 30% of normal, classified as G6PD deficient, where all hemizygous G6PD-deficient males and all homozygous G6PD-deficient females lie, 2) < 70% of normal, the threshold established in the tafenoquine clinical trials for eligibility for receiving tafenoquine, and 3) < 80% of normal, the current World Health Organization threshold for defining intermediates (30–80% of normal activity) (Table 1). The Pointe Scientific assay was used as the gold standard and the 2 × 2 tables from which sensitivity and specificity values cited in Table 1 are provided as Supplemental Files 3 and 4.

**Table 1:** Summary data for the performance of the Trinity Biotech quantitative G6PD assay and the SD Biosensor STANDARD G6PD test compared with the Pointe Scientific quantitative G6PD assay

G6PD diagnostic test	Trinity Biotech quantitative reagent kit	SD Biosensor STANDARD G6PD test		
Study description	Fresh venous blood (K_2_EDTA), PATH, USA	Fresh venous blood (K_2_EDTA), PATH, USA	Fresh venous and capillary blood (both K_2_EDTA), PATH, USA	Frozen venous blood (K_2_EDTA), SMRU, Thailand
No clinical sample (male/female)	183	210	100	150
	Male: 128	Male: 137	Male: 60	Male: 42
	Female: 55	Female: 73	Female: 40	Female: 108
No deficients	21	25	10	54
No intermediates	7	13	8	53
Adjusted PS median (U/g Hb)	9.6	8.89	8.97	6.84
Normal* PS (SD) (U/g Hb)	10.4 (2.4)	9.7 (2.1)	9.3 (1.8)	6.7 (1.1)
Normal* device (SD) (U/g Hb)	11.4 (3.0)‡	10.9 (2.9)†	11.4 (2.4)†	6.7 (1.2)†
Optimal threshold (U/g Hb) equivalent to 30% normal	2.9	2.7	2.7	2.1
% Sensitivity (95% CI)	100.0 (82.4–100.0)	100.0 (95.7–100.0)	100.0 (83.2–100.0)	100.0 (93.4–100.0)
% Specificity (95% CI)	98.2 (94.7–99.6)	97.0 (94.5–98.5)	100 (98.0–100.0)	94.8 (88.3–98.3)
AUC	> 0.99	> 0.99	1	0.99
Optimal threshold (U/g Hb) equivalent to 70% normal	6.7	6.2	6.3	4.8
% Sensitivity (95% CI)	100.0 (86.8–100.0)	95.5 (89.7–98.5)	97.2 (85.5–99.9)	95.0 (88.8–98.4)
% Specificity (95% CI)	100.0 (97.7–100.0)	97.0 (94.5–98.6)	80.5 (73.6–86.3)	81.6 (68.0–91.2)
AUC	1	> 0.99	0.98	0.97
Optimal threshold (U/g Hb) equivalent to 80% normal	7.7	7.1	7.2	5.5
% Sensitivity (95% CI)	89.7 (75.8–97.1)	95.0 (89.5–98.2)	97.8 (88.5–99.9)	96.3 (90.7–99.0)
% Specificity (95% CI)	95.1 (90.2–98.0)	86.3 (81.9–90.1)	63.6 (55.5–71.2)	74.4 (58.8–86.5)
AUC	0.96	0.98	0.95	0.97

AUC = area under the curve; CI = confidence interval; G6PD = glucose-6-phosphate dehydrogenase; K_2_EDTA = dipotassium ethylenediaminetetraacetic acid; PS = Pointe Scientific; SD = standard deviation; SMRU = Shoklo Malaria Research Unit. Confidence intervals are provided in brackets under each sensitivity and specificity value.

* Normal by PS.

† Normal by SD Biosensor STANDARD G6PD test.

‡ Estimates for the Trinity Biotech assay.

### Correlation between the two quantitative laboratory tests for G6PD activity.

**Figure 1.**
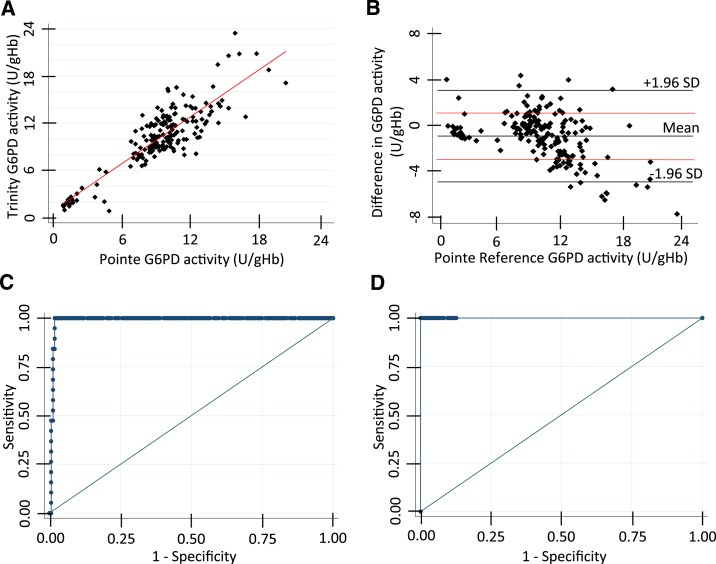
Correlation between two reference quantitative assays for glucose-6-phosphate dehydrogenase (G6PD) activity: Pointe Scientific and Trinity Biotech. (**A**) Linear regression comparing the G6PD values measured on the same specimens by both assays. Glucose-6-phosphate dehydrogenase values normalized for hemoglobin are shown (U/g Hb). (**B**) Bland–Altman plot comparing the two data sets. Lines indicating the mean, 1.96 times the standard deviation (SD), and a difference in 2 U/g Hb in red are shown. (**C**) Receiving operating characteristic curve analysis used to establish optimal threshold values for 30% (left) and (**D**) 70% (right). This figure appears in color at www.ajtmh.org.

The correlation between the Trinity Biotech quantitative G6PD assay and the Pointe Scientific assay was evaluated with 183 clinical specimens, including 21 G6PD-deficient specimens and seven G6PD-intermediate specimens. The sample size was constrained by the availability of the Trinity quantitative assay product that had been discontinued. Both assays were run at 30°C. The same hemoglobin value measured by HemoCue was used to normalize the G6PD activity values measured by both assays. 30°C Trinity G6PD values were correlated to 37°C Pointe Scientific G6PD values. Linear regression gave a squared correlation coefficient (*R*^2^) value of 0.76 and Bland–Altman showed no obvious bias with most specimens falling within an allowable bias of ± 2 U/g Hb for G6PD (Figure 1). Sensitivity and specificity calculated by performing a ROC analysis to identify the optimal values for the 30%, 70%, and 80% thresholds (Table 1, Figure 1B) showed very good concordance in classifying deficient, intermediate, and normal activity between the two assays.

### Analytical performance of the SD Biosensor STANDARD G6PD test: temperature and humidity stress testing.

**Figure 2.**
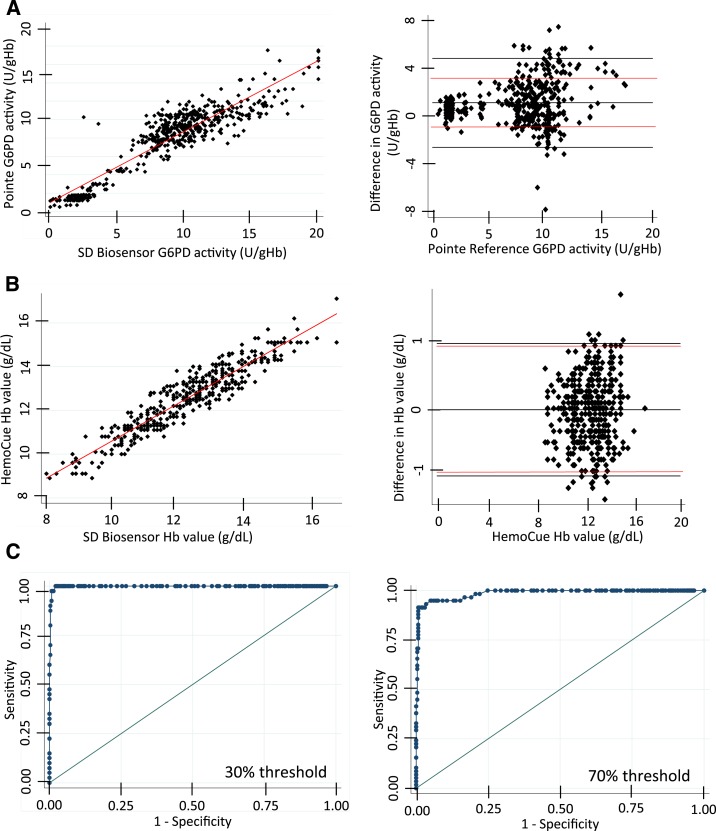
Temperature robustness for glucose-6-phosphate dehydrogenase (G6PD) activity (U/g Hb) and hemoglobin (g/dL) measurement of the STANDARD G6PD test. The STANDARD G6PD test performance was evaluated against the reference assay across multiple temperatures for 210 clinical specimens, including 25 G6PD-deficient and 13 G6PD-intermediate specimens. (**A**) Linear regression plot (left) and corresponding Bland–Altman plot (right) for matched specimens run at 22°C, 32°C 50% humidity, 37°C 50% humidity, and 37°C 75% humidity; a total of 414 data points. Lines indicating the mean, 1.96 times the standard deviation, and a difference in 2 U/g Hb in red are shown on the Bland–Altman plots. (**B**) Linear regression plot (left) and corresponding Bland–Altman plot (right) for the hemoglobin values (g/dL) for the same 414 data points shown in **A** on the STANDARD G6PD test compared with the Hemocue hemoglobin values (used to normalize the reference Pointe Scientific G6PD values). (**C**) Receiving operating characteristic curve analysis for the G6PD values used to establish optimal threshold values for 30% (left) and 70% (right). This figure appears in color at www.ajtmh.org.

The STANDARD G6PD test consists of an analyzer and an assay kit (Supplemental File 2). After running a blood specimen, the analyzer displays hemoglobin concentration (in g/dL) and temperature-corrected G6PD activity normalized for hemoglobin (U/g Hb at 37°C). Two hundred and ten venous blood specimens in K_2_EDTA, including 25 G6PD-deficient specimens and 13 intermediate specimens, were used to verify the ability of the product to correct for temperature. All 210 specimens were run on the benchtop in the laboratory at room temperature, approximately 22°C, and a random subset of 100 samples, including 15 G6PD-deficient and five intermediate, were run in a temperature and humidity controlled chamber at 32°C 50% humidity (Supplemental File 5). No statistical difference was observed between results generated at 22°C and 32°C, and the *R*^2^ value of the combined data sets was 0.81. Two test results (out of 310) corresponding to specimens identified as normal by the reference assay were misclassified as deficient by the STANDARD G6PD test. This misclassification was not observed in repeat testing, and the root cause was not identified. In addition, 10 of the deficient specimens, five intermediates, and 15 normal samples were run at 37°C, 50% humidity and 37°C, 75% humidity to further stress test the STANDARD G6PD test. An analysis combining all test results, including data measured over the full temperature range of 22–37°C, as individual data points and analyzed for correlation and performance against the reference assay (Figure 2) showed good correlation with an *R*^2^ value of 0.85. Hemoglobin measurement on the STANDARD G6PD test showed a tight correlation to the HemoCue, within 1 g/dL Hb and at *R*^2^ of 0.87 across all conditions (Figure 2).

Sensitivity and specificity values, based on optimal thresholds determined by ROC curve analysis and the AUC, showed that the STANDARD test performed equivalently to the Trinity assay when evaluated against the Pointe Scientific assay, despite being stressed over broad environmental operating conditions (Table 1).

### Performance of the SD Biosensor STANDARD G6PD test in matched venous and capillary specimens.

For 100 of the 210 venous specimens, capillary finger stick specimens were also collected in K_2_EDTA vacutainer tubes to test the performance of the STANDARD G6PD test compared with the reference Pointe Scientific G6PD assay normalized by the HemoCue hemoglobin measurement for the venous samples. These specimens included 10 G6PD-deficient and eight intermediate samples. The Pointe Scientific assay only has performance claims for venous specimens, so G6PD values for both venous and capillary samples were compared with the G6PD values of Pointe Scientific venous samples. The hemoglobin-normalized G6PD values from both venous and capillary samples showed good correlation against the Pointe Scientific values with an overall *R*^2^ of 0.85. No bias was observed for either the venous or the capillary specimens (Supplemental File 6).

Venous hemoglobin values from the STANDARD G6PD test were compared with venous hemoglobin values from the HemoCue device and STANDARD G6PD capillary hemoglobin values were compared with HemoCue capillary values. The correlation for the venous hemoglobin measurements was good between the assays, with an *R*^2^ of 0.87. Correlation in hemoglobin values for the capillary samples was initially poor. After 58 samples were run, additional rigorous mixing of the blood samples was introduced before dispensing the aliquot for the assay. Improved correlation was observed for the 42 samples with an *R*^2^ value of 0.64 (Supplemental File 6). The high variability in hemoglobin measurement was not reflected in the hemoglobin-normalized G6PD values for the capillary samples. Good sensitivity was attainable at all thresholds, albeit with a drop in specificity for intermediates, whereby normals by Pointe Scientific were classified as intermediates by the STANDARD test (Table 1).

### Performance for the STANDARD G6PD test on samples from the Thai–Myanmar border population.

**Figure 3.**
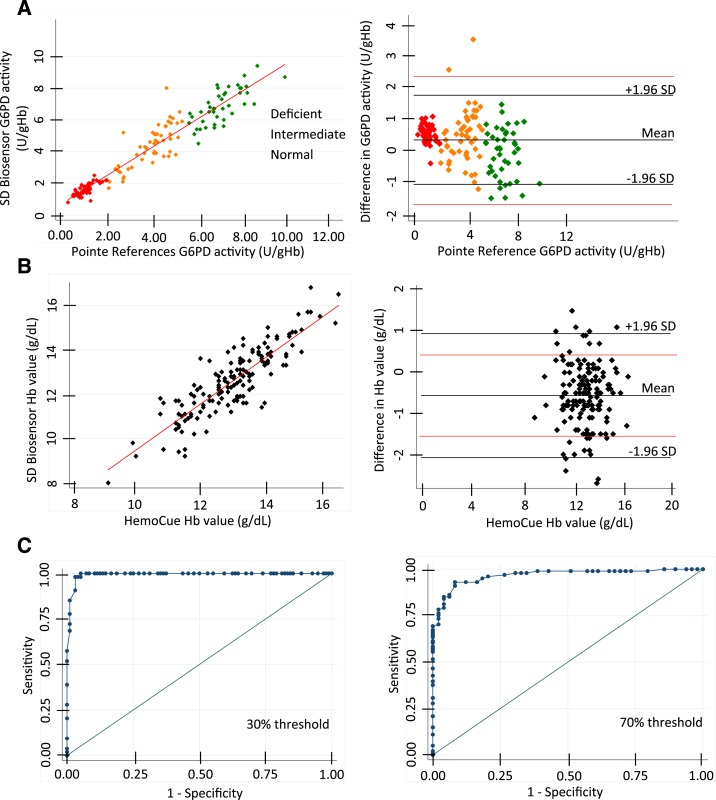
Performance of the STANDARD glucose-6-phosphate dehydrogenase (G6PD) test with frozen blood samples from a Thai–Myanmar border population. STANDARD G6PD test performance was evaluated against the reference assay with 150 clinical specimens: 54 G6PD-deficient (red), 53 G6PD-intermediate (orange), and 43 normal (green) specimens. (**A**) Linear regression plot (left) and corresponding Bland–Altman plot (right) for the G6PD values. (**B**) Linear regression plot (left) and corresponding Bland–Altman plot (right) for the hemoglobin values. Lines indicating the mean, 1.96 times the standard deviation (SD), and a difference in 2 U/g Hb in red are shown on the Bland–Altman plots. (**C**) Receiving operating characteristic curve analysis used to establish optimal threshold values for 30% (left) and 70% (right). This figure appears in color at www.ajtmh.org.

One hundred and fifty frozen, anticoagulated, whole blood, venous samples collected in a previous study were used to evaluate the performance of the STANDARD G6PD test with a geographically distinct population to the previous studies, and in an independent laboratory. The specimens included 54 G6PD-deficient samples (< 30% activity) and 53 with intermediate activity, between 30% and 80% of normal. A good correlation was observed for both normalized G6PD activity and hemoglobin against their respective reference assays, with *R*^2^ values of 0.92 and 0.75, respectively. All G6PD values with the exception of two were within 2 U/g Hb variance of the reference value (Figure 3). Good sensitivity and specificity values were observed for deficients with a slight decrease in specificity for intermediates (Table 1). One of 49 female samples with intermediate G6PD activity levels as determined by the reference assay was misclassified as normal by the STANDARD G6PD test and exhibited greater than 2 U/g Hb variance in G6PD values from the reference assay. This sample had reference assay values of 4.6 U/g Hb and 8.0 U/g Hb on the STANDARD G6PD test. Other misclassifications of females with intermediate activity to normal were all within 2 U/g Hb of the reference value.

### Performance of SD Biosensor unitized G6PD control reagents.

SD Biosensor also provides unitized high and low G6PD controls to support quality assurance of the STANDARD G6PD test. The performance of the quality control reagents provided by SD Biosensor was also evaluated across two devices and under two temperature and humidity conditions. The reproducibility over five replicates gave a coefficient of variance of under 6.8% in all measurements except for the low control G6PD values, where the variance was higher because of the small values (Table 2).

**Table 2:** Performance of quality control reagents provided by SD Biosensor

	Low control				High control			
	Device 1		Device 2		Device 1		Device 2	
	Mean	CV (%)	Mean	CV (%)	Mean	CV (%)	Mean	CV (%)
	(SD)		(SD)		(SD)		(SD)	
	Range		Range		Range		Range	
Room temperature								
Hb (g/dL)	11.2	5.2	11.3	6.5	15.6	3.4	14.8	5.2
	(0.58)		(0.73)		(0.53)		(0.77)	
	10.4–12.3		10.1–12.5		14.7–16.6		13.5–15.6	
G6PD (U/g Hb)	0.5	17.7	0.4	15.7	7.9	3.5	8.2	6.8
	(0.1)		(0.07)		(0.3)		(0.6)	
	0.4–0.7		0–0.5		7.4–8.4		6.9–9.1	
35°C, 50% Humidity								
Hb (g/dL)	11.3	3.4	10.9	3.1	14.6	2.9	15.2	3.4
	(0.38)		(0.34)		(0.42)		(0.52)	
	10.6–11.9		10.3–11.3		14.0–15.3		14.4–16.1	
G6PD (U/g Hb)	0.6	20.8	0.5	56.3	8.8	3.2	8.5	3.8
	(0.1)		(0.3)		(0.3)		(0.3)	
	0.4–0.8		0–0.8		8.5–9.2		7.9–9.0	

CV = coefficient of variation; G6PD = glucose-6-phosphate dehydrogenase; SD = standard deviation. Five replicates were run for each condition on two SD Biosensor analyzers.

## Discussion

G6PD deficiency is the most common human genetic condition, with potential lifelong clinical implications.^1,2,23^ Glucose-6-phosphate dehydrogenase deficiency is highly prevalent in malaria-endemic populations, where resources are limited.^24^ Primaquine and tafenoquine, both 8-aminoquinolines, when given with blood-stage antimalarials can cure patients of *P. vivax* but may also cause clinically relevant hemolysis in patients with decreased G6PD activity. Ensuring safe, radical curative treatment requires the availability of G6PD deficiency rapid diagnostic tests that are easy-to-use and affordable.^10^ Qualitative tests developed in recent years attempt to address these needs but they do not accurately identify females heterozygous for G6PD deficiency with intermediate G6PD activity.^12,15,25^ Some of these females may be at risk of hemolysis, if exposed to the doses of 8-aminoquinolines required to eliminate hypnozoites.^16,26^ A point-of-care test that can measure G6PD activity normalized by hemoglobin concentration, with a broad operating temperature range could address this limitation. As per the World Health Organization prequalification technical specifications for in vitro diagnostics to measure G6PD activity, such a test must demonstrate the ability to discriminate from normal, G6PD-deficient (< 30%), and G6PD-intermediate (30–80% of normal) activity.^27^ The performance of the test at the inclusion threshold (> 70%) used in the clinical trials for tafenoquine is also relevant, as this will be used to inform treatment with this drug.^9^

In this study, two tests for G6PD deficiency were evaluated against the gold standard laboratory quantitative assay for G6PD activity (Pointe Scientific) combined with the gold standard hemoglobin assay (HemoCue). One evaluation compared the Pointe Scientific quantitative assay against the Trinity Biotech G6PDH Quantitative kit, a laboratory-based quantitative assay for G6PD activity that was widely used as a reference gold standard for the evaluation of other G6PD products until production was suspended in 2017. Both the Trinity and Point Scientific assays require temperature-regulated spectrophotometers, a separate hemoglobin measurement, and then manual calculation of G6PD activity normalized for hemoglobin. In the other evaluation, the SD Biosensor STANDARD G6PD point-of-care test was compared with the Pointe Scientific assay. The STANDARD G6PD test concurrently measures G6PD activity and hemoglobin, functions over a broad operating temperature, and provides G6PD activity in international units within 2 minutes without any user calculations required.

The G6PD activity values in the two reference assays were only equivalent when comparing the G6PD activity at 30°C on the Trinity assay with those corrected to 37°C on the Pointe Scientific assay. Both assays used the same hemoglobin measurement. Good sensitivity and specificity were observed for discriminating G6PD-deficient specimens (< 30% normal activity) and G6PD-intermediate specimens (either < 70% or < 80%) using the Pointe Scientific assay as the gold standard, despite an *R*^2^ value of 0.76.

The STANDARD G6PD test demonstrated equally good performance with a panel of 210 whole-blood venous specimens from African American volunteers under multiple temperature and humidity operating conditions, ranging from 22°C to 37°C and up to 75% humidity. The capillary G6PD values (U/g Hb) correlated well with the gold standard−matched venous G6PD values for all specimens, despite poor correlation between the two hemoglobin measurements performed on capillary specimens. This indicates that hemoglobin was measured accurately but that deviation from the HemoCue values was a result of genuine differences in the amount of hematocrit drawn into the assay. It is apparent that the measurement of hemoglobin from the same blood sample on which the G6PD assay is performed contributes to the accuracy of the normalized G6PD value (U/g Hb). The STANDARD G6PD test also performed well when evaluated in Thailand with a diverse sample set in a second laboratory, different G6PD genotypes, and good coverage across the full clinically relevant G6PD activity range. For this sample set, the normal G6PD activity was low, both on the gold standard assay and the STANDARD G6PD test, compared with the U.S. sample sets, most likely resulting from the freeze–thaw process; however, this did not affect the concordance between the two tests. The data presented here also has limitations with respect to establishing the robustness of this promising product. Further clinical studies are required in near-patient or intended settings, with larger samples sizes for fresh non-anticoagulated capillary specimens. Future studies should also evaluate the usability of the product by the intended users (not the expert laboratory staff used for this study) and their ability to accurately interpret and record the test results.

The results raise several considerations when assessing new tests for G6PD deficiency. A perfect correlation between two quantitative tests for enzyme activity is very challenging to achieve.^13^ In addition, there is significant interlaboratory variability in running the same reference assay, which further impacts the ability to establish a perfect correlation between the two assays.^28–30^ To minimize interlaboratory variability, PATH established a proficiency panel composed of frozen samples. A critical question to resolve is whether robust universal G6PD activity thresholds (in U/g Hb) can be established to discriminate G6PD-deficient (< 30% normal) and intermediate (< 70% or < 80% of normal) activity, or whether it will be necessary to determine these in each setting. This will require more data from multiple settings and more clinical specimens than described here.

## Conclusion

Although automated and near-patient quantitative tests for G6PD deficiency have been previously described, many of which rely on fluorescence, none are widely available.^18,31^ The SD Biosensor STANDARD G6PD test represents a true near-patient product that provides quantitative G6PD results, bringing the possibility of managing all patients—males, females, and neonates—equally with respect to G6PD deficiency, minimizing risk of hemolysis, and improving health outcomes. Specifically, it represents a true opportunity to greatly increase access to radical cure of *P. vivax* malaria, and thus support malaria elimination, and provides an opportunity to address a gender gap in our understanding of G6PD epidemiology and its clinical implications.

## Supplementary Material

Supplemental files
