# Supplementary material for: Evaluation of a Novel Quantitative Test for Glucose-6-Phosphate Dehydrogenase Deficiency: Bringing Quantitative Testing for Glucose-6-Phosphate Dehydrogenase Deficiency Closer to the Patient
Source: Am J Trop Med Hyg. 2018 Oct 22;100(1):213–21. doi: 10.4269/ajtmh.18-0612 (PMC6335905; doi:10.4269/ajtmh.18-0612)
Supplement: Supplementary file 1 [file tpmd180612.SD1.pdf]

Supplemental Files for Evaluation of a novel quantitative test for G6PD deficiency: bringing quantitative testing for G6PD deficiency closer to the patient

Supplemental File 1. Flow diagram for recruitment and testing for the US based study.

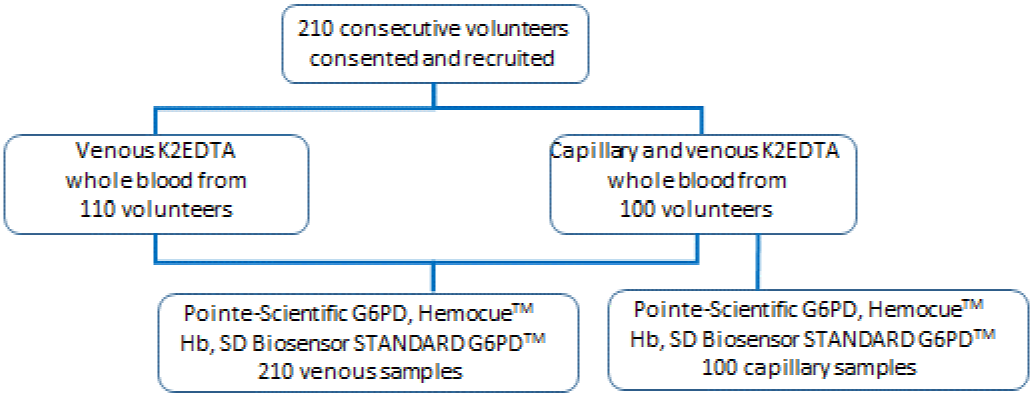

Supplemental File 2. Illustration of the SD Biosensor STANDARD G6PD test. The test is composed of an instrument and a kit. The kit includes a single-use test device, two sample collectors per test device, one extraction buffer vial per test device, one code chip per kit, and instructions for use.

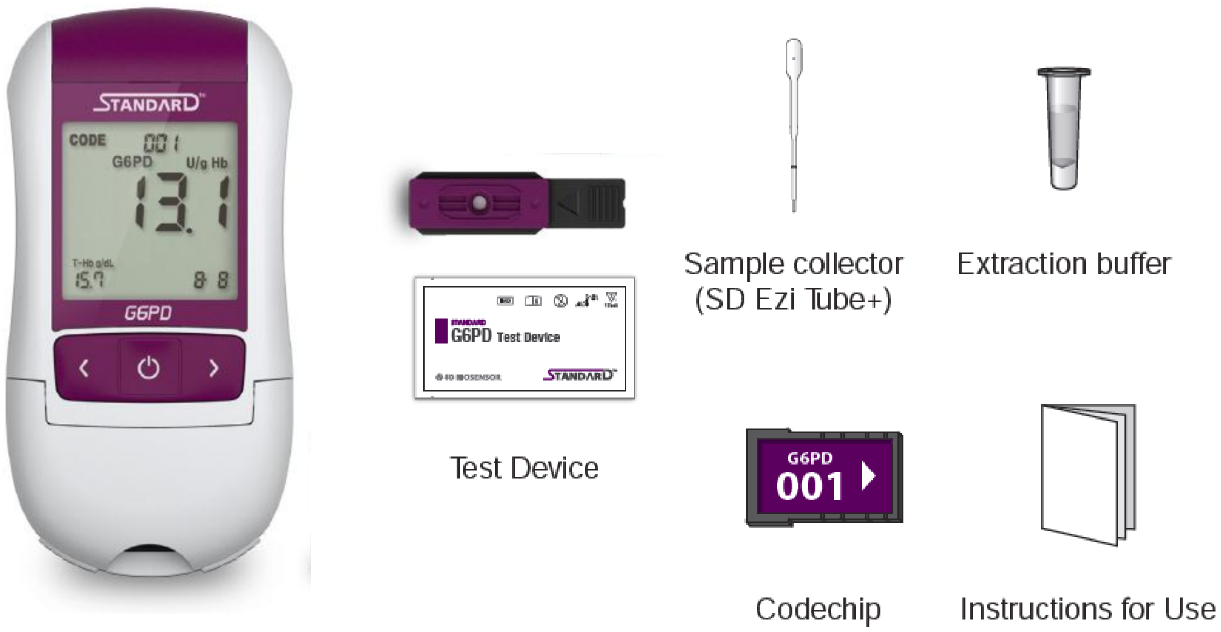

Supplemental File 3.
2 x 2 tables for determination of sensitivity and specificity for the Trinity quantitative G6PD test with the Pointe Scientific as gold standard. True positives (TP) and True negatives (TN) refer to the Pointe Scientific determinations. Positives (P) and negatives (N) correspond to the Trinity values. *Totals (T) are also provided.*

**Deficient (<30%)**			
	**TP**	**TN**	***T***
**P**	19	3	*22*
**N**	0	161	*161*
T	*19*	*164*	*183*

**Deficient/intermediate (<70%)**			
	**TP**	**TN**	***T***
**P**	26	0	*26*
**N**	0	157	*157*
T	*26*	*157*	*183*

**Deficient/intermediate (<80%)**			
	**TP**	**TN**	***T***
**P**	35	7	*42*
**N**	4	137	*141*
T	*39*	*144*	*183*

Supplemental File 4.
2 x 2 tables for determination of sensitivity and specificity for the SD Biosensor STANDARD G6PD test with the Pointe Scientific as gold standard. True positives (TP) and True negatives (TN) refer to the Pointe Scientific determinations. Positives (P) and negatives (N) correspond to the STANDARD G6PD test values. *Totals (T) are also provided.*

US venous study

**Deficient (<30%)**			
	**TP**	**TN**	***T***
**P**	84	10	*94*
**N**	0	320	*320*
T	*84*	*330*	*414*

**Deficient/intermediate (<70%)**			
	**TP**	**TN**	***T***
**P**	105	9	*114*
**N**	5	295	*300*
T	*109*	*304*	*414*

**Deficient/intermediate (<80%)**			
	**TP**	**TN**	***T***
**P**	115	40	*155*
**N**	6	253	*259*
T	*121*	*293*	*414*

Thailand frozen venous study

**Deficient (<30%)**			
	**TP**	**TN**	***T***
**P**	54	5	*59*
**N**	0	91	*91*
T	*0*	*96*	*150*

**Deficient/intermediate (<70%)**			
	**TP**	**TN**	***T***
**P**	96	9	*105*
**N**	5	40	*45*
T	*101*	*49*	*150*

**Deficient/intermediate (<80%)**			
	**TP**	**TN**	***T***
**P**	103	11	*114*
**N**	4	32	*36*
T	*107*	*43*	*150*

Supplemental File 5.

Temperature robustness for G6PD and hemoglobin measurement (g/dL) of the STANDARD G6PD test. The STANDARD G6PD test performance was evaluated against the reference assay across multiple temperatures for 210 clinical specimens, including 25 G6PD-deficient and 13 intermediates. (A) Linear regression plot (left) and corresponding Bland–Altman plot (right) for G6POD measurements for 210 specimens run at 22°C (in blue) and 100 matched specimens at 32°C 50% humidity (in orange); a total of 310 data points. Lines indicating the mean, 1.96 times the standard deviation (SD), and a difference in 2IU/gHb in red are shown on the Bland–Altman plots. (B) Linear regression plot (left) and corresponding Bland–Altman plot (right) for the hemoglobin values comparing the STANDARD G6PD test to the Hemocue hemoglobin measurement (g/dL Hb) for the same 310 G6PD data points. Lines indicating the mean, 1.96 times the standard deviation (SD), and a difference in 1 g/dL in red are shown on the Bland–Altman plots.

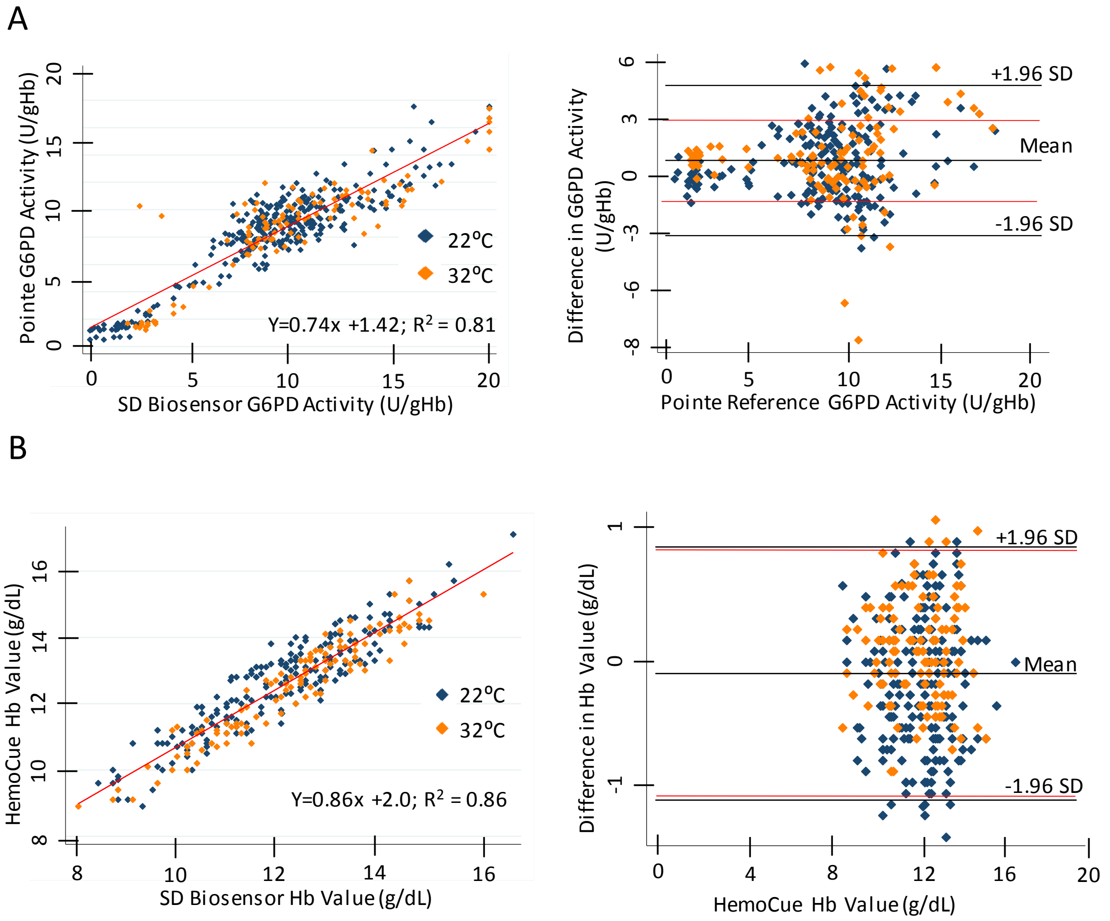

Supplemental file 6 (figure). Performance of the STANDARD G6PD test with matched capillary and venous anticoagulated (K_2EDTA) specimens. STANDARD G6PD test performance was evaluated against the reference assay at 22°C for 100 specimens for which matched venous and capillary blood specimens had been collected. Results from venous specimens are indicated in blue diamonds and capillary specimens in orange. (A) Linear regression plot (left) and corresponding Bland–Altman plot (right) for G6PD activity for all 200 measurements. (B) Linear regression plot (left) and corresponding Bland–Altman plot (right) for hemoglobin measurement for all capillary specimens on the STANDARD G6PD test compared to HemoCue on capillary samples (n=100 blood samples). (B) All 100 results are shown, though the linear regression was fitted only to the 42 samples (filled diamonds) once thorough mixing was introduced. Lines indicating the mean, 1.96 times the standard deviation (SD), and a difference in 1 g/dL in red are shown on the Bland–Altman plots.

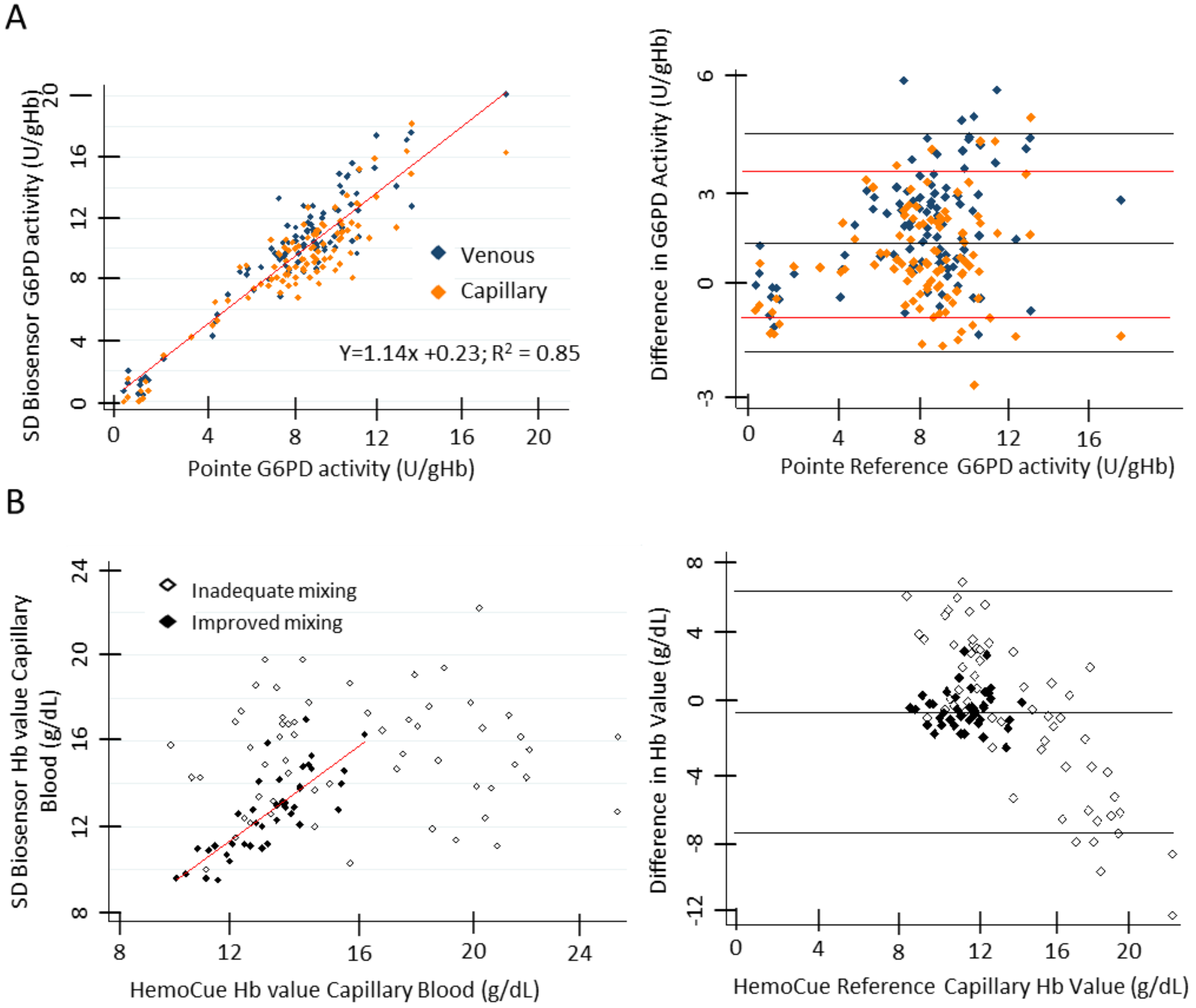

(A) Linear regression plot (left) and corresponding Bland□Altman plot (right) for 210 specimens run at 22°C (in blue) and 100 matched specimens at 32°C 50% humidity (in orange); a total of 310 data points.
